# Tailored Telemonitoring in patients with heart failure: results from a multicentre randomized controlled trial (the TEHAF-study)

**Published:** 2012-06-15

**Authors:** Josiane Boyne, Hubertus Vrijhoef, Harry Crijns, Fred Nieman, Gerjan Deweerd, Johannes Kragten, Anton Gorgels

**Affiliations:** Maastricht University Medical Centre, The Netherlands; The University Tilburg, The Netherlands; Maastricht University Medical Centre, The Netherlands; Maastricht University Medical Centre, The Netherlands; Orbis Medical Concern, The Netherlands; Atrium Medical Concern, The Netherlands; Maastricht University Medical Centre, The Netherlands

**Keywords:** heart failure, telemonitoring, tailored care, RCT, education

## Abstract

**Background:**

Recent increasing prevalence of heart failure (HF) patients leads to an increasing burden to the health care system. Consequently, there is a need for innovative strategies to reduce HF hospitalizations.

**Methods:**

We performed a multicentre randomized controlled trial to test the hypothesis that telemonitoring in patients with HF, by means of the Health Buddy^®^ system (HB), will reduce HF hospitalizations and number of contacts with caregivers as compared to care as usual (CAU) during 1 year follow-up, from October 1, 2007, through December 31, 2008.

**Results:**

Among 382 patients—197 in the HB and 185 in the CAU-group—226 (59%) were male, mean age was 71.5 (SD 11.2), 45.5% being ≥75 years of age; 57% of the patients were in NYHA HF class 2, 40% in class 3 and 3% in class 4. Both study groups were similar for demographic and clinical characteristics. Mean time to first heart failure related hospitalization was 161 days for the intervention group and 139 days for the usual-care group; hospitalizations occurred in 18 (9.1%) compared to 25 patients (13.5%) respectively (Kaplan–Meier p=0.151, hazard ratio 0.65, CI 0.35–1.17). Combined endpoint of heart failure admission and all cause mortality was similar for both groups (Kaplan–Meier p=0.641 hazard ratio 0.89, CI 0.69–1.83). Cox regression analysis disclosed an important interaction between group assignment and heart failure duration, p=0.007, OR=0.983, CI 0.970–0.995 indicating a significant decrease in heart failure hospitalizations in the intervention group if heart failure duration was <18 months, p=0.026, hazard ratio 0.26, CI 0.07–0.94. Contacts with the heart-failure-nurse were mean 1.36 (range 0–11) in the intervention group vs. 1.74 (0–8) in the usual-care group (Mann-Whitney p<0.001). Mortality was 18 (9.1%) in the intervention-group and 12 (6.5%) in the usual-care-group (Mann–Whitney p=0.34, Cox-regression analysis p=0.82).

**Conclusion:**

Telemonitoring tends to reduce heart failure admissions and decreases contacts with specialized nurses. If heart failure duration is <18 months heart failure admissions and readmissions are significantly reduced.

